# A Wideband High-Gain Microstrip Array Antenna Integrated with Frequency-Selective Surface for Sub-6 GHz 5G Applications

**DOI:** 10.3390/mi13081215

**Published:** 2022-07-29

**Authors:** Husam Alwareth, Imran Mohd Ibrahim, Zahriladha Zakaria, Ahmed Jamal Abdullah Al-Gburi, Sharif Ahmed, Zayed A. Nasser

**Affiliations:** 1 Microwave Research Group (MRG), Centre for Telecommunication Research & Innovation (CeTRI), Fakulti Kejuruteraan Elektronik dan Kejuruteraan Komputer (FKEKK), Universiti Teknikal Malaysia Melaka (UTeM), Hang Tuah Jaya, Durian Tunggal 76100, Malaysia; p021820004@student.utem.edu.my (H.A.); zahriladha@utem.edu.my (Z.Z.); alwajeehzaid@yahoo.com (Z.A.N.); 2 Faculty of Engineering and Technology, Multimedia University (MMU), Ayer Keroh 75450, Malaysia; sharifqyousef@gmail.com

**Keywords:** wideband array antenna, frequency-selective surface (FSS), sub-6 GHz, 5G, high gain, stopband

## Abstract

This paper presents a wideband and high-gain rectangular microstrip array antenna with a new frequency-selective surface (FSS) designed as a reflector for the sub-6 5G applications. The proposed antenna is designed to meet the US Federal Communications Commission (FCC) standard for 5G in the mid-band (3.5–5 GHz) applications. The designed antenna configuration consists of 1 × 4 rectangular microstrip array antenna with an FSS reflector to produce a semi-stable high radiation gain. The modeled FSS delivered a wide stopband transmission coefficient from 3.3 to 5.6 GHz and promised a linearly declining phase over the mid-band frequencies. An equivalent circuit (EC) model is additionally performed to verify the transmission coefficient of the proposed FSS structure for wideband signal propagation. A low-cost FR-4 substrate material was used to fabricate the antenna prototype. The proposed wideband array antenna with an FSS reflector attained a bandwidth of 2.3 GHz within the operating frequency range of 3.5–5.8 GHz, with a fractional bandwidth of 51.12%. A high gain of 12.4 dBi was obtained at 4.1 GHz with an improvement of 4.4 dBi compared to the antenna alone. The gain variation was only 1.0 dBi during the entire mid-band. The total dimension of the fabricated antenna prototype is 10.32 λ_o_ × 4.25 λ_o_ ×1.295 λ_o_ at a resonance frequency of 4.5 GHz. These results make the presented antenna appropriate for 5G sub-6 GHz applications.

## 1. Introduction

Over the next five years, data traffic is expected to expand sevenfold due to an increase in mobile users, smartphones, and internet of things connections, and network speed increases and cellular video consumption. The bandwidth-hungry video, which makes up 78 percent of mobile traffic, will place an even greater burden on mobile networks [1,2,3]. Due to these challenges and the ultra-high transmission rate (peak data of 10 Gbps), the low latency (on a millisecond), extremely high traffic density (106/km^2^), and super high mobility (500 km/h), the 5G mobile communication system will be deployed in the future, employing terahertz frequencies [4,5]. The new 5G radio access networks are expected to support numbers of connections simultaneously while operating across a wide range of frequencies [6]. In order to enable 5G, FCC divided the key range into a low band (up to 1 GHz), mid band of 3.5 GHz (under 6) and high band (mmWave) [7,8]. The mmWave offers high-capacity data rates of more than two gigabits per second while the low band covers the 5G and medium bands well. To reach the goal of ultra-fast data rates, it is clear that the 5G mmWave spectrum should be used. However, significant obstacles must be overcome before mmWave mobile communications are implemented. Before the completion of mmWave technology for 5G communication, sub-6 GHz will emerge as the 5G technology in the near future [9,10]. High data speeds may be transferred over a wide range of distances using sub-6 GHz 5G transmission. It can be used in urban and rural areas [11]. In sub-6 GHz, Malaysian Communications and Multimedia Commission (MCMC) has allocated frequency ranges of 3.3–3.8 GHz as the priority spectrum for 5G deployment [12].

Recently, some standard electromagnetic simulation techniques have been proposed for antenna engineering enhancements, such as those for highly reflective index [13], metal-dielectric metal (MDM) [14], surface plasmon resonance absorber [15], double plasmon-induced transparencies in aperture-coupled metal-dielectric-metal (MDM) [16], and highly sensitive refractive index based on metamaterial [17].

Many efforts have been made to improve the antenna design performance for 5G applications. Researchers aim to design an antenna with a compact size, low cost, and high performance. Thus, this antenna could be applied for indoor and outdoor base stations. To improve the radiation of the antenna design, different design techniques have been applied. In [18], a hybrid-mode antenna was presented for sub-6 GHz communication. The proposed antenna was compact and composed of a slotted rectangular patch, a feeding dipole, and a balun, where these three modes were excited in a shared patch. This hybrid-mode antenna achieved an impedance bandwidth of 56.87% and an average gain of approximately 8.00 dBi in the operating frequency band of 3.0–5.0 GHz. Despite these advantages, the proposed antenna design feeding is quite complex. In [19], three mutual coupling reduction techniques were proposed to design a compact phased array using a planar inverted-E antenna (PIEA) consisting of 4 elements and operating at 5.7 to 6.4 GHz. Although this antenna achieved a gain of 8.36 dBi and a scanning angle of ±70 degrees, this antenna suffers from high cross-polarization. In [20,21,22,23,24,25,26], slot antennas have the advantages of a simple design, high performance, and small size. These attractive benefits of the slot antenna have directed the attention of researchers to apply these benefits in antenna design. Thus, the whole communication system’s size and performance would be enhanced. These designs can achieve a wide impedance bandwidth. However, their achieved gain is poor. In [27], a study of the C-shaped slot of a microstrip patch antenna on a 7 × 7 array reactive impedance surface (RIS) was conducted. This antenna was designed to have miniaturization and circular polarization. Although the proposed c-shaped slot antenna can achieve an impedance bandwidth of 46.52% (3743–5976 MHz), the antenna gain is limited to 6.8 dBi. In [28], a wideband Y-shaped slot monopole antenna with a meandered slot fed by a coplanar waveguide (CPW) was proposed. Multiple resonance modes were achieved due to the advantage of the meandered slot, a slotted Y-shaped element, and a trident-shaped feed strip. Moreover, a compact λ/4 spaced four-element array was fabricated based on a single element monopole antenna design. This antenna design could provide a wide bandwidth of 41.8%. However, the gain was limited to 2 dBi, and it suffered high cross-polarization. For sub-6 GHz 5G application, a defected ground-structured (DGS) antenna with a stub-slot configuration is presented in [29]. A simple stub-slot configuration was studied in the patch antenna to achieve a dual-band frequency response. Further, the 2 × 2 electronic band gap (EBG) technique has been studied in the ground plane for impedance bandwidth enhancement. This antenna design can achieve an impedance bandwidth of 12.49% at 3.532 GHz and 4.49% at 6.835 GHz. However, the gain is limited to approximately 3.5 dBi and suffers from back radiation.

A conventional microstrip rectangular array antenna with a slotted square ring of a frequency-selective surface (FSS) as a reflector for sub-6 GHz 5G applications is presented in this study. This antenna is designed to cover the standardized 5G mid-band by achieving a broad bandwidth and high semi-stable gain. The proposed antenna obtains a fractional bandwidth of 51.12% (3.5–5.8 GHz), which is considerably more comprehensive than recent studies. Meanwhile, the efficiency reaches 77.5% (3.3–5.6 GHz) and a high gain of 12.4 dBi is obtained. An equivalent circuit (EC) model is designed using computer simulation technology (CST) and successfully compared with the advanced design system (ADS) for FSS transmission coefficient (S21) validations.

## 2. Configuration of the Antenna Design

**Figure 1:**
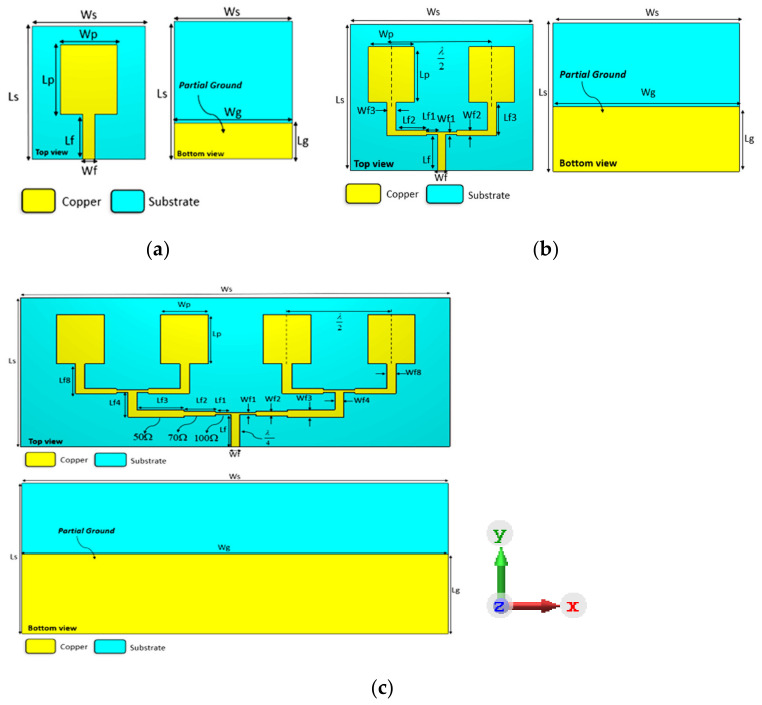
Development of the wideband microstrip array antenna: (**a**) single element, (**b**) 1 × 2 array antenna, (**c**) 1 × 4 array antenna.

The Computer Simulation Technology (CST) software was used to simulate the proposed array antenna configuration. The fundamental features of the proposed array antenna, such as the frequency resonance, reflection coefficient, bandwidth, gain, and polarization factors, are contemplated for the antenna design. The low-cost FR4 substrate is used to simulate and fabricate the finalized array antenna along with the FSS reflector, and it has a relative permittivity of 4.4, thickness (h) of 1.6 mm, and dielectric loss (tan (δ)) of 0.019. The design evolution of the proposed array antenna is presented in Figure 1. The first step starts with designing a single patch radiating element, followed by 1 × 2 and 1 × 4 array patches for a better wideband impedance bandwidth. After, the 1 × 4 array antenna is integrated with a slotted square ring (SSR) of the FSS reflector to obtain high gain and a wide bandwidth.

The proposed 1 × 4 array antenna is designed at 4.5 GHz, which has a geometry size denoted as the substrate width (Ws) × substrate length (Ls) × substrate height (h) of 136 × 55 × 1.6 mm^3^ and 166 × 66 × 1.6 mm^3^ for FSS reflector. The FSS reflector is placed under the array antenna with an airgap S = λ/4. The final optimum parameters of the array antenna and FSS reflector are shown in Table 1 and Table 2, respectively.

### 2.1. Antenna Design Process

The rectangular microstrip patch antenna is designed at the 4.5 GHz resonant frequency utilizing the mathematical equations of the microstrip antenna as a conventional reference design in order to model the structure of the 1 × 2 and 1 × 4 array antennas. The dimensions of this basic rectangular patch antenna, denoted as (W) and (L) for the width and the length of the patch, can be determined using the equation from [30]:

(1) $$W=\frac{c}{2f_{r}\sqrt{\frac{\varepsilon_{r}+1}{2}}}\;L_{eff}=\frac{c}{2f_{r}\sqrt{\varepsilon_{eff}}}$$

(2) $$\varepsilon_{eff}=\frac{\varepsilon_{r}+1}{2}+\frac{\varepsilon_{r}-1}{2}{\left[1+12\frac{h}{w}\right]}^{-\frac{1}{2}}$$

(3) $$\Delta L=0.412h\frac{\left(\varepsilon_{eff}+0.3\right)\left(\frac{w}{h}+0.264\right)}{\left(\varepsilon_{eff}-0.258\right)\left(\frac{w}{h}+0.8\right)}$$

where c= velocity of light, $f_{r}=centre$ frequency, $\varepsilon_{r}=$ permittivity of dielectric material, $\varepsilon_{eff}=$ effective dielectric constant, and ΔL is the length extension. For the feedline dimensions, when (w/d)>2, it can be calculated using:

(4) $$\frac{w}{d}=\frac{2}{\pi }\left[B-1-\ln(2B-1)+\frac{\varepsilon_{r}-1}{2\varepsilon_{r}}\times \left[\ln(B-1)+0.39-\frac{0.61}{\varepsilon_{r}}\right]\right]$$

(5) $$B=\frac{377\pi }{2Z_{o}\sqrt{\varepsilon_{r}}}$$

**Figure 2:**
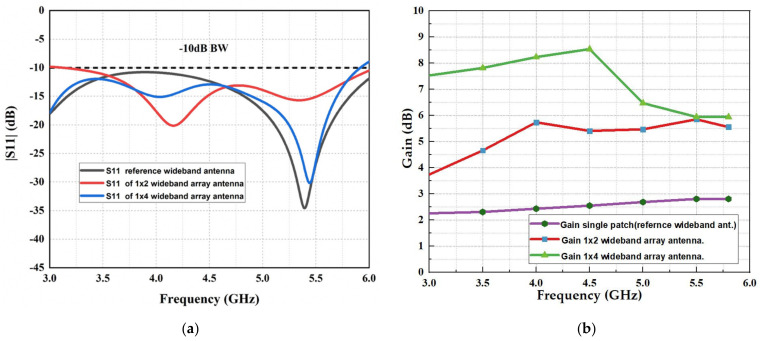
Simulated results of wideband microstrip array antenna development: (**a**) reflection coefficient (S11), (**b**) realized gain.

With respect to the antenna diagrams illustrated in Figure 1, these designs introduce the microstrip rectangular patch array antenna development. The first design shown in Figure 1a started from a basic rectangular patch as a reference for the array antenna design. The total antenna dimension for the single patch is 30 mm × 35 mm × 1.6 mm printed on the FR4 substrate, which is fed by an impedance 50 Ω feedline as discussed in [30]. It is designed at a resonant frequency of 4.5 GHz with a partial ground of Lg = 9.84 mm. The partial ground is utilized to attain a wide bandwidth. To improve the antenna’s performance, the 1 × 2 array antenna consists of two rectangular radiating patches connected by a two-way power divider of a quarter-wave transformer impedance, which has the dimensions of 60 mm × 48 mm × 1.6 mm, as shown in Figure 1b. Meanwhile, the last modification for antenna gain enhancement is to design a 1 × 4 array antenna that consists of four radiation patches with a spacing between the patch elements of λ/2 half of the wavelength at the resonance frequency of 4.5 GHz, which are connected to a four-way power divider of quarter-wave transformer impedance using FR-4 substrate, where it has dimensions of 136 mm × 55 mm × 1.6 mm as shown in Figure 1c. The simulation results of the reflection coefficient (S11) and realized gain of the development of the wideband array antenna are presented in Figure 2, which shows the impact of increasing the radiating elements. It can be observed that in Figure 2a, the single patch element resulted in a reflection coefficient below −10 dB at the frequency operating from 2.5 to 6.1 GHz. Figure 2b shows a realized gain of approximately 2.4 dBi across the bandwidth. At the same time, the reflection coefficient starts operating from 3.3 to 6 GHz for the 1 × 2 array antenna. The realized gain obtained alternates between 4.5 and 5.5 dBi across the operating spectrum. Meanwhile, the 1 × 4 array antenna received an operating bandwidth of 2.4 to 5.9 GHz, and the realized gain is observed around 7.5 to 8.5 dBi at the operating frequency of 3 to 4.5 GHz while it is decreasing for the rest bandwidth because of impedance matching of the feedlines and mutual coupling between the radiating patches.

### 2.2. Parametric Study for the 1 × 4 Array Antenna

**Figure 3:**
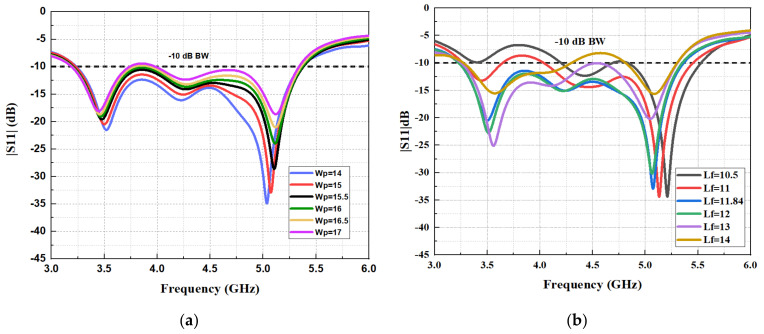
Reflection coefficient (S11) and bandwidth study for the dimensions change of: (**a**) width of the patch (Wp) and (**b**) feedline length (Lf).

Figure 3 presents the parametric study for the width of the radiating patch and the length of the 50 Ω impedance feedline for the 1 × 4 array antenna in order to achieve optimum impedance matching and reduce the mutual coupling between the radiating rectangular patches. The parametric study was performed using the parameter sweep function in CST 2020 Studio Suite^®^ simulation software. One of the essential principles of rectangular patch antennas is that it radiates from the width of the rectangular antenna patch. The patch length parameter was chosen for the ideal design when Lp was fixed at 18.25 mm. The effect of changing the width of the rectangular patch denoted as (Wp) can be observed in the reflection coefficient response in Figure 3a. A poor reflection coefficient response is observed when the width of the rectangular patch is increased because the spacing between the radiating patches produces mutual coupling. The optimum value for the patch width was chosen as Wp = 15 mm, where the reflection coefficient response is below −10 dB from 3.3 to 5.3 GHz. Another parametric study for the reflection coefficient response was performed for the length of the feedline denoted as (Lf) presented in Figure 3b. Mismatch of the impedance occurred when changing the length of the feedline, where at Lf = 10.5 mm and Lf = 14 mm, the reflection coefficient response was above –10 dB. Hence, the optimum length for the feedline was chosen as Lf = 11.84 mm at a resonance frequency of 4.5 GHz, where the operating bandwidth is from 3.3 to 5.3 GHz.

## 3. Design of the FSS Reflector

**Figure 4:**
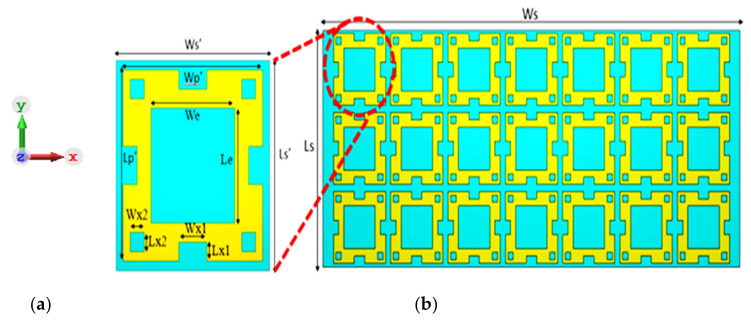
Geometry of the proposed FSS reflector: (**a**) SSR unit cell and (**b**) 3 × 7 SSR arrays.

To model an appropriate array antenna for wideband 5G sub-6 GHz applications, an FSS with an array of conductive unit cells can be used as the bottom layer. The FSS would resonate at a particular frequency band, and it will be invisible to others. Thus, as it performed a band stop filter, it produced a reflective surface. The FSS, when integrated with a PEC ground plane, reflects the transmitted waves outside the resonance band. This FSS design is used as the ground of the antenna that can provide an almost constant gain across the desired frequency band. The initial parameters taken to design the square unit cell are Ws’ = 1.46 λ_o_, h =0.106λ_o_, and Ls’ = 1.46 λ_o_, where λ_o_ corresponds to the resonance frequency of the free space wavelength at 4.5 GHz. The reflection phase response is between −90° and 90°, which is dominated by the SSR dimensions parameters of the unit cell. The requirement for a wideband unit cell substrate is a wider Q factor. Therefore, the FR4 substrate was chosen with a dielectric constant of (ε_*r*_ = 4.4) at 4.5 GHz, the loss tangent of 0.019, and a thickness of h = 1.6 mm. After parametric analysis, the optimized values obtained to achieve the desired frequency band are shown in Table 2. The SSR unit cell with the 3 × 7 SSR array configuration design is shown in Figure 4.

### 3.1. Parametric Study of the FSS Unit Cell

**Figure 5:**
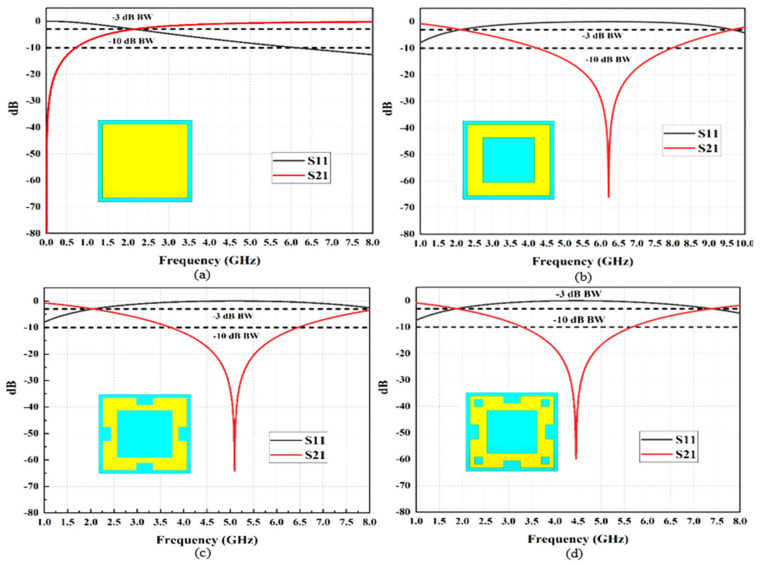
Simulated results of the S11 and S21 of the proposed FSS unit cells: (**a**) square metallic patch, (**b**) square ring, (**c**) square ring with edge rectangular slot, and (**d**) slotted square ring.

The optimum metallic slotted square ring (SSR) can be utilized to achieve broad bandwidth response characteristics in FSS when all the fourth portions of the square ring are equal to the λ/4 quarter wavelength. The implementation of slots will attain a comprehensive bandwidth response, where the outer ring of the square patch reacts to the high resonance frequency and the inner ring of the square patch responds to the low resonance frequency, which has a stopband performance, as shown in Figure 5b. The advantage of producing slots is that it can enhance the bandwidth of the resonance frequency. Therefore, it can be observed that the resonance frequency is shifted by introducing the rectangular edge slot and the square slots, which leads to the creation of a stopband response from 3.8 to 6.5 GHz, as shown in Figure 5c. The floquet port features in CST were used to simulate the SSR unit cell of the FSS reflector. The modeled SSR promised wide stopband features operated from 3.3 to 5.6 GHz and a net bandwidth of 2.3 GHz, with a −60 dB of return loss performance at 4.49 GHz, as shown in Figure 5d.

**Figure 6:**
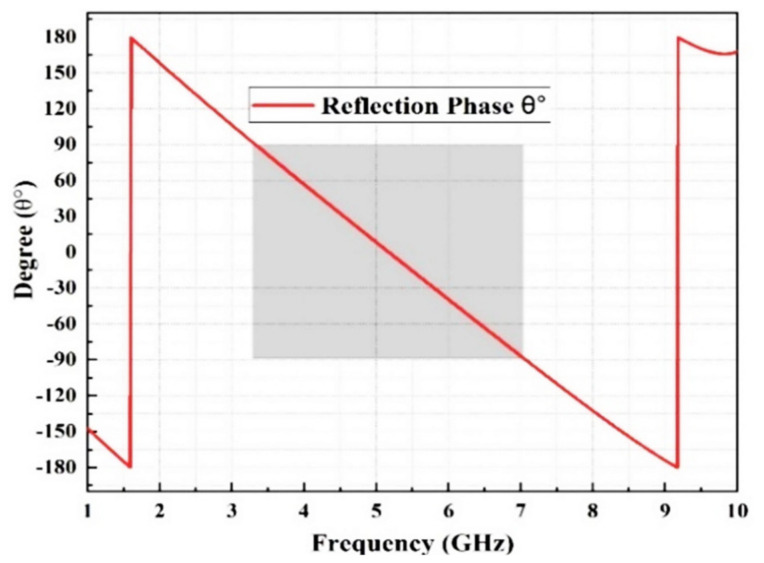
Simulated reflection phase.

In Figure 6, the highlighted grey square shows the reflection phase response from 90° to −90°, which is demonstrated by the FSS unit cell operating at a frequency of 3.2 to 6.8 GHz, as suggested in [31]. Hence, it is essential to produce declining phase reflection at the desired operating frequency for the active stop band filter, which can react as a magnetic conductor for the vertical incident waves produced by the array antenna. This decline over the wideband increases the gain at its maximum when the radiated waves (from the antenna) are in phase with the incoming waves from the FSS [31].

### 3.2. Equivalent Circuit (EC) of FSS

**Figure 7:**
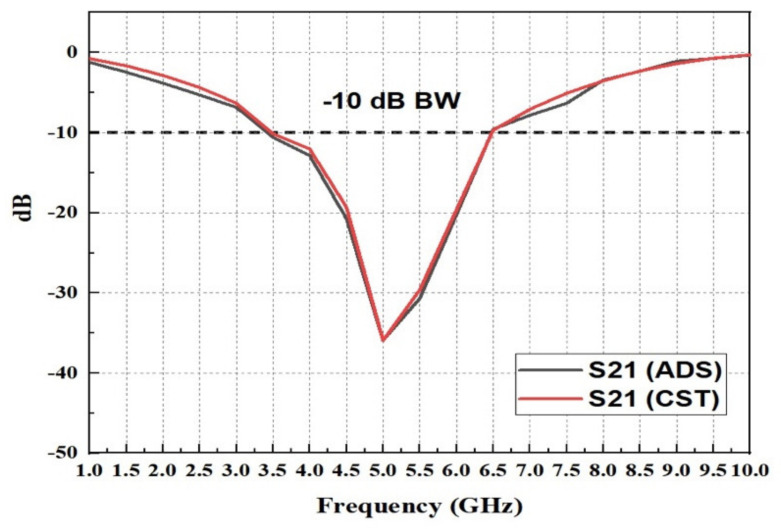
S21 comparison of the equivalent circuit result with simulation.

In this study, the equivalent circuit (EC) was designed utilizing advanced Design System (ADS) software. The EC model was developed and scheduled based on the designed FSS configuration and delivered two important lumps components, which are: (1) inductance (L) and (2) capacitance (C) circuit lumps. Figure 7 compares the simulation results of ADS and CST to determine if a matching circuit represents the desired unit cell. It is evident that the ADS and CST simulations provide almost identical results, although with a narrower bandwidth and greater peak resonance.

**Figure 8:**
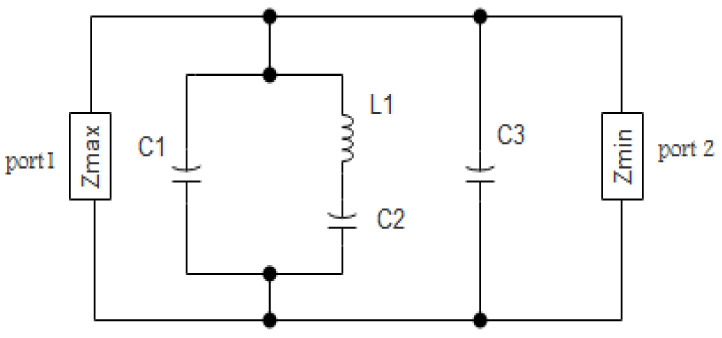
EC model of the FSS.

The wideband unit cell acts as a resonant band pass filter in this configuration; hence, its equivalent circuit (EC) model derived from [32,33] is shown in Figure 8, where capacitance (C1), (C2), and (C3), and inductance (L1) can be estimated from:

(6) $$Z_{FSS}=\frac{\left[\omega^{2}C_{1}-\left(1-\omega^{2}C_{1}L_{1}\right)\left(1-\omega^{2}C_{2}\right)-\left(1-\omega^{2}C_{2}L_{1}\right)\left(1-\omega^{2}C_{3}\right)\right]}{j\omega C_{1}\left(1-\omega^{2}C_{2}\right)\left(1-\omega^{2}C_{3}\right)}$$

where:

(7) $$C_{1}=\frac{\varepsilon_{0}\varepsilon_{r}LW}{2h}$$

(8) $$L_{1}=\frac{1}{\omega^{2}C_{1}}$$

(9) $$C_{2}=\frac{1}{L_{1}\omega_{p2}^{2}}$$

(10) $$C_{3}=\frac{\left(\omega_{p2}^{2}-\omega_{z1}^{2}\right)\left(\omega_{p2}^{2}-\omega_{z2}^{2}\right)}{-L_{1}\omega_{p2}^{2}\omega_{z1}^{2}\omega_{z2}^{2}}$$

The swivel (pole) and zeros pair were the values of the variables ωp1 and ωp2. The neural impedance (Z_o_) was equal to 50 Ω. Meanwhile, the value of ωp1 was assumed to be zero [34]. These variables were calculated using Equations (7)–(10). The RLC values were estimated using the previously mentioned equations reported in Table 3.

### 3.3. Parametric Study for the Number of Unit Cells and the Air Gap for FSS Reflector with the Array Antenna

After improving the reflection and transmission coefficients of the proposed FSS unit cell, an extensive parametric study was performed by varying the quantities of unit cells of the FSS reflector and the air gap between the array antenna and the FSS reflector to determine the optimum values for attaining high gain for the entire bandwidth.

**Figure 9:**
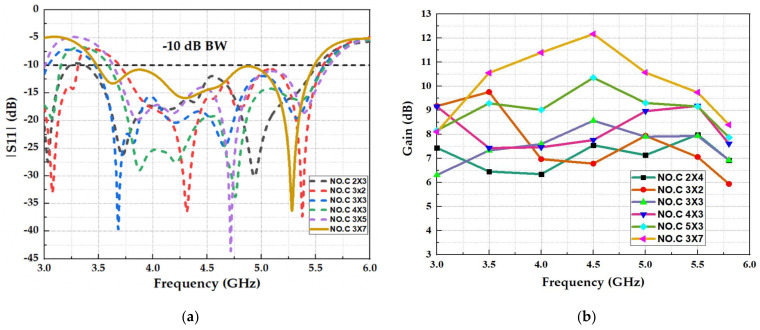
Simulated parametric study for the number of unit cells: (**a**) reflection coefficient (S11) and (**b**) realized gain.

The total size of the antenna is limited by the dimensions of the FSS unit cells. As a result, an extensive investigation of the influence of the size of the FSS unit cell on the radiated waves of the array antenna was performed by varying the quantities of the FSS unit cells (NO.C), as shown in Figure 9.

The outcomes of this analysis are displayed in Figure 9. Figure 9a comprises the reflection coefficient of the antenna for various quantities of cells (NO. C), which models the size of the FSS, indicating that the matching band of the antenna is primarily influenced by the region of the FSS that is located directly under the antenna. In other words, when the FSS proportions surpass the antennas, the antenna’s bandwidth becomes independent of the FSS size.

The reflective behavior of the wideband FSS is calculated by assuming infinite FSS proportions, which cannot be recognized in practice where finite-size configurations are required. Nevertheless, infinite proportions can be approximated with considerable quantities of FSS cells. Nonetheless, the size of the FSS impacts on the antenna’s radiation behavior, as shown in Figure 9b, across a parametric study of the antenna peak gain, showing that as the number of cells increases, the gain also grows over the entire wide band. A smaller reflector can be used at the expense of the achieved gain.

**Figure 10:**
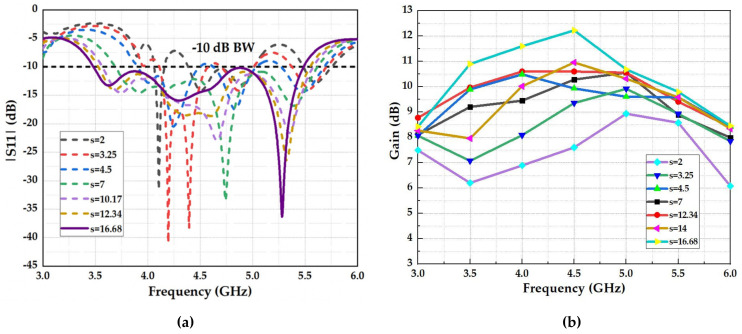
Simulated parametric study for the air gap between the array antenna and FSS reflector: (**a**) reflection coefficient (S11) and (**b**) realized gain.

Another important parametric study was conducted for the spacing (S) between the FSS reflector and the wideband array antenna, as shown in Figure 10. As expected, the FSS influences the marching band of the antenna as when “S” grows, the antenna’s bandwidth expands, as presented in Figure 10a. Likewise, the effect of this parameter on the radiation properties of the antenna is depicted in Figure 10b, where the gain across a wide band is estimated for various matters of “S”. This shows that the gain varies differently over the frequency, which can be described by the fact that the phase change counted by “S” is an operation of the frequency.

In summary, we can infer that the working band, stability, and the value of the highest gain of the antenna positively affect how far the antenna is from the FSS reflector. The reflection coefficient (S11) achieved an appropriate response and high stable gain across the band when S= 16.68 mm, equivalent to λ/4, as shown in Figure 10a,b.

## 4. Simulation and Measurement Results

**Figure 11:**
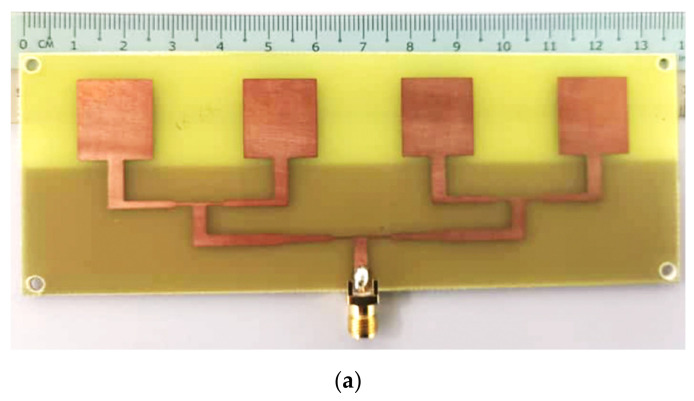

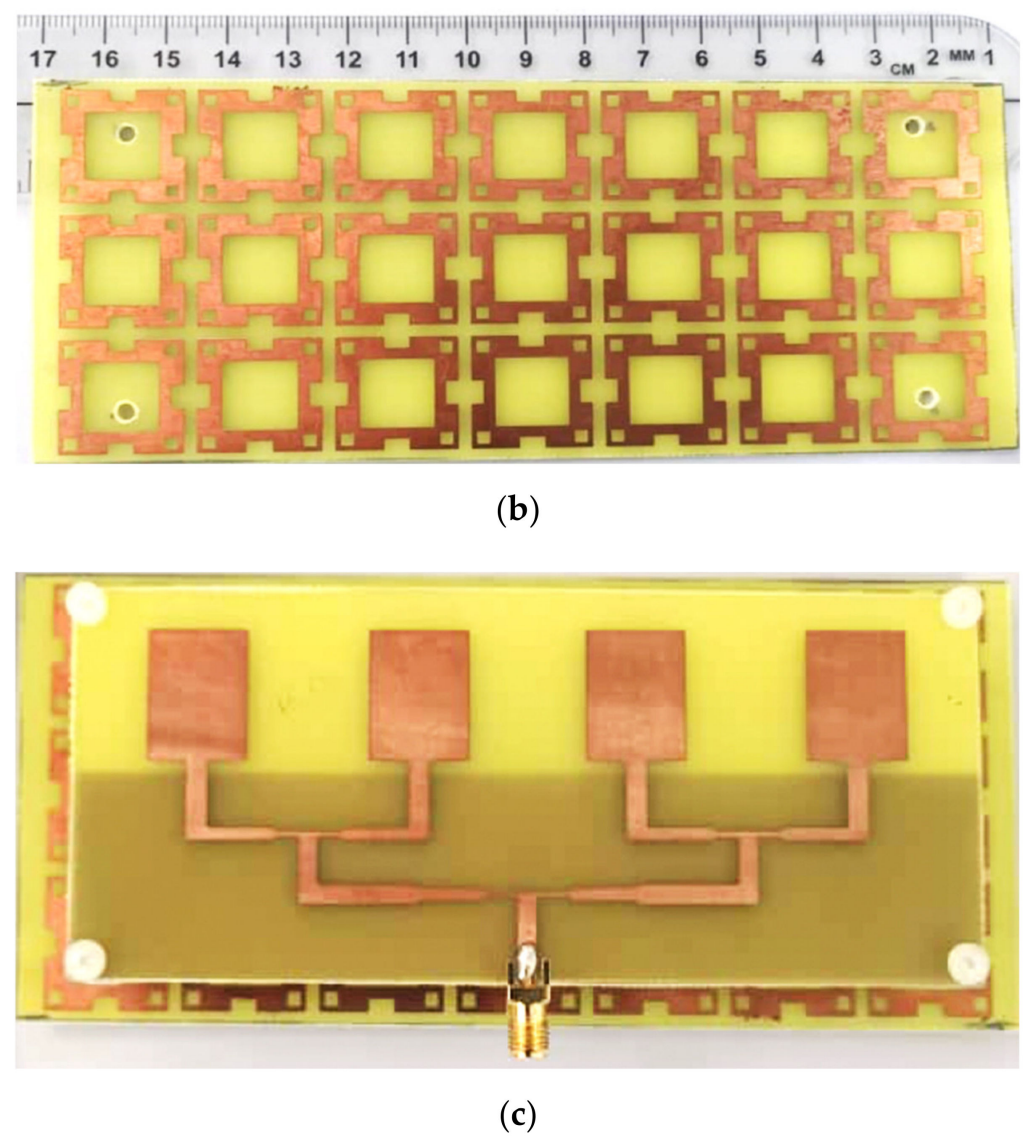
Photograph of the wideband array antenna with the measurement setup: (**a**) prototype of the 1 × 4 array antenna, (**b**) prototype of the 3 × 7 FSS reflector, and (**c**) integration of the array antenna with the FSS reflector.

The proposed array antenna design with an FSS reflector was fabricated using a low-cost FR-4 substrate material, as shown in Figure 11a,b, respectively. The entire physical dimension of the antenna was 160 mm × 66 mm × 20.08 mm, which is equivalent to 10.32 λ_o_ × 4.25 λ_o_ × 1.295 λ_o_ free space dimensions at 4.5 GHz. The utilization of plastic spacers to hold the array antenna and FSS reflector at air gap λ/4 is shown in Figure 11c. The measurements of the array antenna with and without the FSS reflector were set in free space as a receiver side to collect the RF signals transmitted by the horn antenna to compare the enhancement generated by the FSS reflector, where the horn antenna has a gain of 10 dBi at 4.5 GHz. Meanwhile, the horn antenna transmits input powers (Pin) from 0 to 20 dBm.

**Figure 12:**
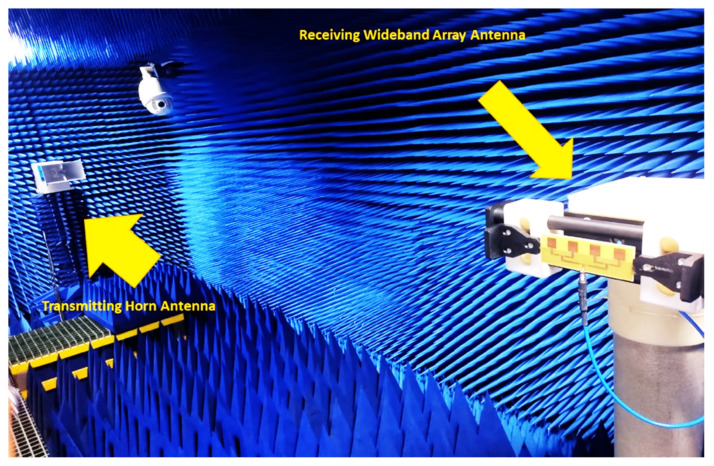
Radiation pattern and gain measurement setup of the antenna.

The experimental setup of the antenna was performed in free space in an anechoic chamber to measure the radiation pattern and the gain, as shown in Figure 12. The reflection coefficient (S11) was measured using the Vector Network Analyzer (VNA). In addition, the gain was calculated utilizing the Friis equation [35].

### 4.1. Reflection Coefficient (S11) Results

**Figure 13:**
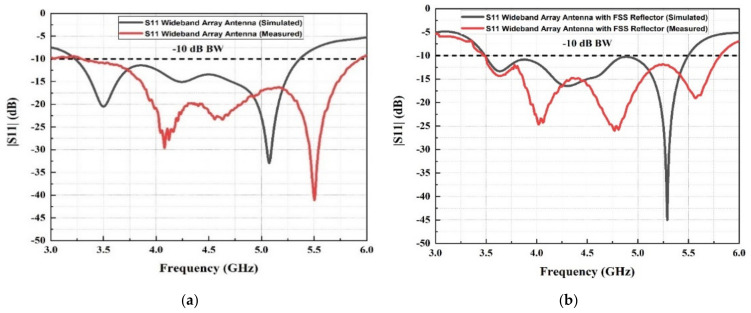
Simulated and measured reflection coefficient (S11) for (**a**) the wideband array antenna without the FSS reflector and (**b**) the wideband array antenna with the FSS reflector.

Figure 13 shows a comparison between the simulation and measurement results of the reflection coefficients response for the wideband array antenna with and without the FSS reflector. The simulation results were obtained using CST software, whereas the measurement results were obtained using Vector Network Analyzer (VNA). It can be observed that the −10 dB reflection coefficient response for the array antenna without an FSS reflector covers the frequency band for simulation from 3.25 to 5.25 GHz and measurement from 3.3 to 5.9 GHz, as illustrated in Figure 13a. At the same time, the reflection coefficient response produced from the simulation and measurement of the wideband array antenna integrated with the FSS reflector covers the sub-6 GHz band, as it can be observed that the reflection coefficient was obtained from 3.5 to 5.5 GHz for the simulation results. In comparison, it covers from 3.5 to 5.8 GHz for the measurement results, achieving a bandwidth of 2.3 GHz and a fractional bandwidth of 51.12%, as shown in Figure 13b. Therefore, it is evident that the FSS reflector acts as a passband filter when it is integrated with the array antenna. The simulated and measured outcomes differ due to inaccuracies when manufacturing using manual cutting tools in addition to the inhomogeneous dielectric constant of the substrate [36]. The discrepancies between the simulations and measurements are also influenced by the SMA connector losses in practice, as an ideal connector is modelled in simulations. In addition, the amount of power fed into the antenna during the measurements is also affected by how the epoxy is applied to galvanically connect the SMA connector and fabric [37].

### 4.2. Realized Gain, Efficiency, and Radiation Pattern Results

**Figure 14:**
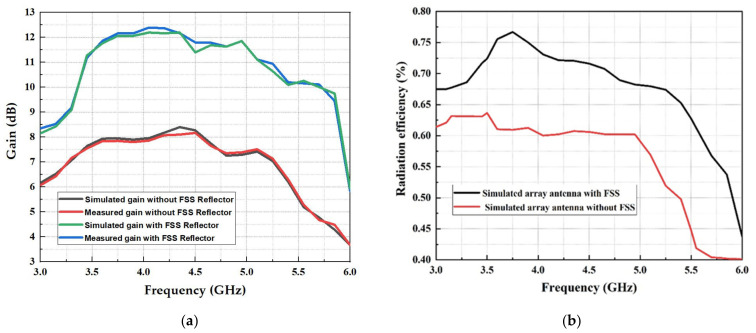
(**a**) Comparison of the simulated and measured gain of the wideband array antenna with and without the FSS reflector; (**b**) simulated efficiency of a wideband array antenna with and without the FSS reflector.

The gain measurement and the efficiency simulation radiation of the reported array antenna with and without an FSS reflector are shown in Figure 14. The measurement setup for the gain, which is illustrated in Figure 12, was carried out in the anechoic chamber at Microwave Research Group Laboratory (MRG) in Universiti Teknikal Malaysia Melaka (UeTM). To measure the gains of the fabricated antenna, the transmitting antenna was demonstrated by a horn antenna (Tx). In contrast, the receiving antenna was shown by the fabricated array antenna with and without the FSS reflector (Rx), where the distance between the (Tx) antenna and (Rx) antenna was 1.5 m.

The comparison of the simulated and measured results of the realized gain for the array antenna with and without an FSS reflector is shown in Figure 14a. It can be observed that a semi-stable gain was achieved for the array antenna with an FSS reflector, where the integration of the FSS reflector improved the gain within the operating frequency range of 3.5 to 5.8 GHz around 4.4 dBi compared to the measured array antenna without the FSS reflector. This denotes the advantages of the proposed FSS reflector, which can enhance the reflected waves of the array antenna. The highest measured gain achieved by the fabricated antenna was 12.4 dBi at 4.1 GHz, whereas the lowest gain achieved was 10 dBi, with an approximately obtained gain of 11.4 dBi at 3.5 GHz. The gain was maintained between 11.4 and 12.4 dBi, with 1.0 dBi of variation in the entire mid-band. Moreover, the gain fluctuation is associated with the difference in the impedance matching at various operating range frequencies. For the radiation efficiency response shown in Figure 14b, it can be observed that the array antenna with an FSS reflector achieved a maximum efficiency of 77.5%. The array antenna without an FSS reflector achieved a maximum efficiency of 63%, which makes the proposed antenna a great candidate for antenna engineering enhancement.

**Figure 15:**
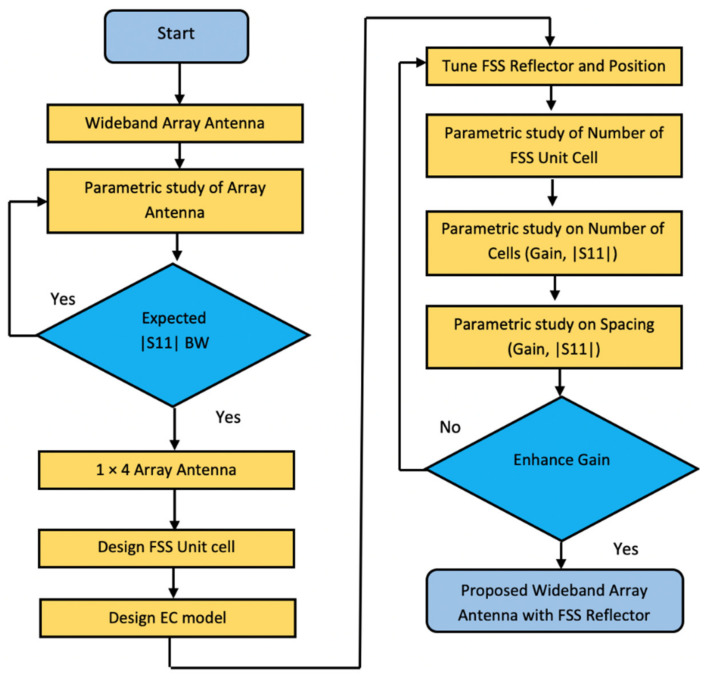
The flowchart for developing the proposed array antenna with FSS.

The methodology of the proposed wideband array antenna advancement and optimization procedure is illustrated in Figure 15 through a flowchart. In this flowchart, the presented array antenna structure and its parameters, such as the number of unit cells (NO.C) and the variation of the spacing (S) parameters playing a pivotal part in optimizing the antenna, are defined to obtain the expected high-gain wideband array antenna within the wide operating frequency.

**Figure 16:**
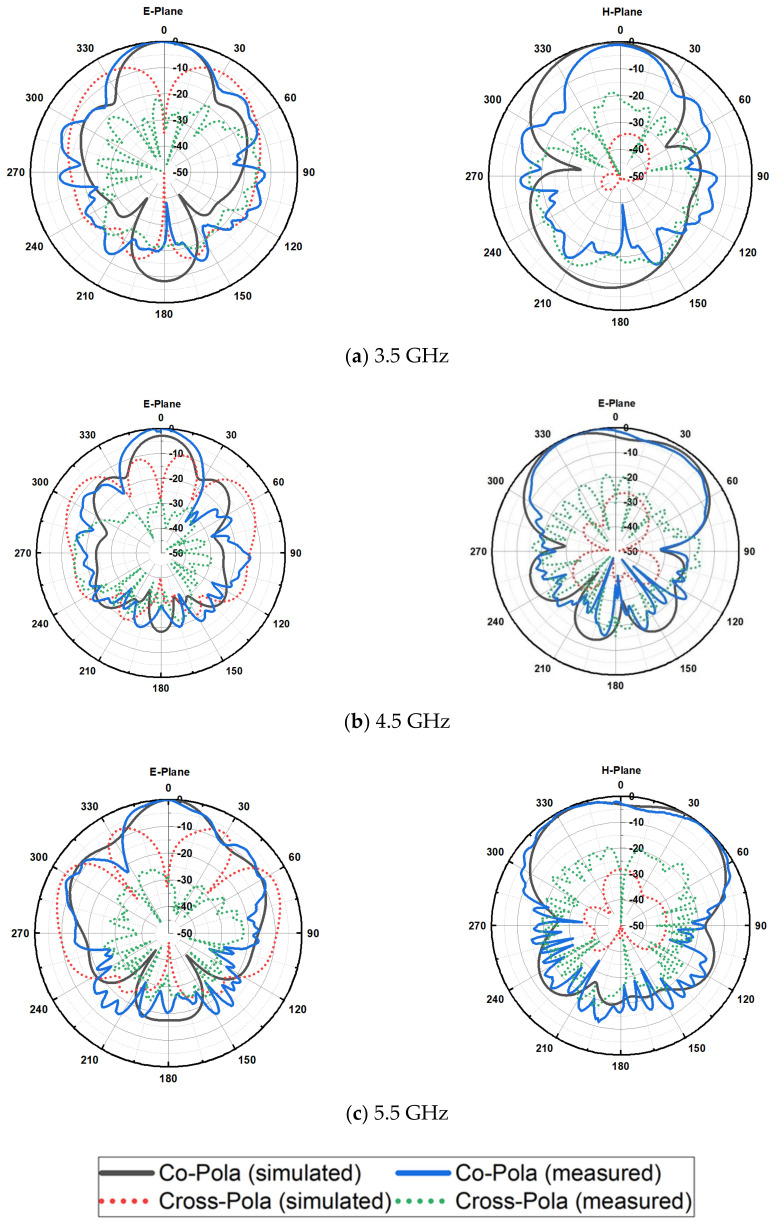
Simulated and measured E-field and H-field radiation patterns showing co-polarization and cross-polarization of the wideband array antenna with the FSS single layer reflector at: (**a**) 3.5, (**b**) 4.5, and (**c**) 5.5 GHz.

A comparison between the CST simulated and anechoic chamber measured results of the radiation patterns in the electrical field (E-plane) and magnetic field (H-plane) for the array antenna with FSS reflector at 3.5, 4.5, and 5.5 GHz is illustrated in Figure 16. A rational agreement between the simulation and measurement of the co-polarization and cross-polarization results can be observed. The array antenna is linearly polarized, which has, at 3.5 GHz, a co-polarization of 0 and −0.117 dB at the angle θ = 0° for the simulation and measurement of the E-plane, respectively. The cross-polarization obtained is −34.79677 and −29.444 dB for the simulated and measured patterns at the same frequency and angle for the E-plane. Moreover, for the simulated and measured H-plane co-polarization, it obtained −0.41801 and −0.81 dB and cross-polarization of −34.7 and −21.89 dB at the angle θ = 0°, respectively. Moreover, the side lobes introduced distortion in the radiation pattern. However, the FSS reflector can maintain high gain due to its ability to absorb diffracted waves of the partial ground plane. At 4.5 GHz, a high cross-polarization is introduced because of the dimensions of the feeding line of the quarter-wave transformer to the approached wavelength. The obtained cross-polarization for the E-plane for the simulated and measured values is −27.08 and −28.99 dB, respectively. At the same time, the simulated and measured co-polarization achieved −2.85 and 0 dB at the angle θ = 0° for both cases. It can be observed that the back lobe at 4.5 GHz is minimized by approximately −9 dB compared to the back lobe at 3.5 GHz, which shows that the propagated waves are reflected for the realization of the linear radiation pattern and the same case for the 5.5 GHz simulation and measurement of co- and cross-polarization for the E-plane and H-plane.

**Figure 17:**
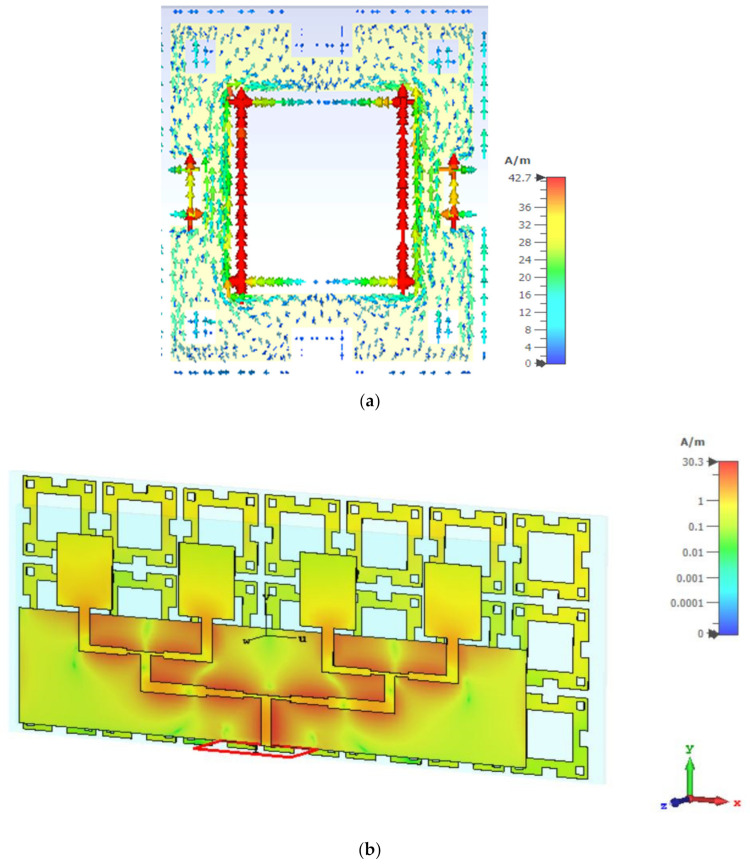
Current density distributions at 3.5 GHz for: (**a**) the FSS unit cell and (**b**) array antenna with the FSS reflector.

To discuss the effect of the FSS reflector operation on the array antenna, the current distribution is proposed in Figure 17. It is revealed that the exited current flow is more energetic in the inner edge of the FSS unit cell, as displayed in Figure 17a. On the other hand, a strong current density distribution can be observed when the array antenna is integrated with the FSS reflector because it acts like a ground and when the current flow is saturated, as shown in Figure 17b.

The characteristics of the proposed array antenna with an FSS reflector are compared in terms of the design size, metamaterial integration, operating frequency range, fractional bandwidth, peak gain, and efficiency with past studies in Table 4. It is obvious that the proposed wideband antenna has a wide operating bandwidth and high realized gain at the expense the antenna size. The antenna asserted a good wideband -10 dB impedance response, and the configuration had no external decoupling network to achieve isolation. By integrating the FSS reflector, the array antenna achieved a high gain of 12.4 dBi, with an acceptable radiation efficiency of about 77.5%. These attractive features make the proposed design suitable for 5G sub-6 GHz applications.

## 5. Conclusions

A high-gain wideband array antenna with an FSS reflector for mid-band 5G applications was examined in this study. Comprehensive parametric studies on the antenna and FSS to reach the optimum design configurations were clearly discussed. The equivalent circuit model was also presented and verified with the important antenna engineering software: CTS and ADS. The suggested wideband array antenna with the integration FSS reflector attained a wide fractional bandwidth of more than 51.12% and a radiation efficiency of 77.5%. Integration of the proposed FSS reflector with an array antenna achieved a semi-stable gain of 12.4 dBi at 4.1 GHz and 11.4 dBi at 3.5 GHz. The gain was kept between 11.4 and 12.4 dBi with 1.0 dBi variations in the entire mid-band. The total array antenna size was 10.32 λ_o_ × 4.25 λ_o_ × 1.295 λ_o_ at a center frequency of 4.5 GHz. In conclusion, these results of the reported wideband array antenna with an FSS reflector show good potential for use in the 5G sub-6 GHz applications.

## Figures and Tables

**Table 1:** Optimum parameters for the 1 × 4 array antenna.

Parameter	Value (mm)	Parameter	Value (mm)
Ws and Wg	136	Wf3	2.745
Ls	55	Lf3	15
Wf	2.745	Wf4	3.038
Lf	11.84	Lf4	9.25
h	1.6	Wf8	3.038
t	0.035	Lf8	11
Lf1	5	Lp	18.25
Wf1	0.79	Wp	15
Wf2	1.78	Lg	29
Lf2	10	S	16.88

**Table 2:** Optimum parameters for the slotted square ring (SSR) of the FSS reflector.

Parameter	Value (mm)	Parameter	Value (mm)
Ws (array)	160	W × 1	4
Ls (array)	66	L × 1	2
Ws (unit cell)	22	W × 2	2
Ls (unit cell)	22	L × 2	2
h	1.6	We	12
t	0.035	Le	12
Wp	20	Lp	20
Ws (array)	160	W × 1	4

**Table 3:** RLC parameters of the ECC.

Circuit Parameter	Values
*C*_1_	66.88 *fF*
*C*_2_	60.21 *fF*
*L*_1_	17.45 *pH*
*C*_3_	3.4 *fF*
Z_o_	50 Ω

**Table 4:** Optimum parameters for the slotted square ring (SSR) of the FSS reflector.

Ref.	Size in (λ_o_^3^)	Design Technique	Frequency (GHz)	FBW (%)	Peak Gain (dBi)	Metamaterial Integration	Radiation Efficiency
[7]	1.26 × 1.22 × 0.623	Five circles with cutting the inner and outer radius	(3.5–5)	34%	2.07	no	null
[21]	0.984 × 0.912 × 0.652	C-shape slot loaded patch antenna with reactive impedance surface (RIS)	(3.74–5.97)	28.90%	6.8	yes	null
[38]	0.257 × 0.257 × 0.06	Sub array antenna with differential feeding	(3.4–3.6)	5.71%	8	no	null
[39]	11.82 × 12.1 × 1.324	1 × 8 hexagonal shaped antenna	(3.5)	----	6.9	no	null
[40]	2.76 × 0.75 × 0.13	Dipole array antenna	(3.30–3.71) (4.79–5.17)	11.7% 7.6%	13.5 15.1	no	80%
#This Work	10.32 × 4.25 × 1.295	Microstrip array Integrated with FSS	(3.5–5.8)	51.12%	12.4	yes	77.5%
